# Bilateral central retinal arterial obstruction following head trauma: A very rare case report

**DOI:** 10.4103/0301-4738.73731

**Published:** 2011

**Authors:** Saumendranath Ghose, Parida Subhabrata

**Affiliations:** Department of Ophthalmology, SCB Medical College and Hospital, Cuttack, Orissa - 753 001, India

**Keywords:** Bilateral central retinal artery obstruction, head injury, embolism

## Abstract

A 30-year-old patient presented at our outpatient department with complaints of severe loss of vision in both eyes following a head injury six days back. He also had a fracture at left side of the mandible and a few bruises over the left cheek. External ocular examination revealed subconjuctival hemorrhage in the left eye and bilateral sluggishly reacting pupils. Fundus examination showed white- out retina and a cherry red spot at the macula in both eyes. A clinical diagnosis of bilateral central retinal arterial obstruction (CRAO) was made which was later confirmed by fundus fluorescence angiography. Bilateral CRAO is a rare disease usually found in patients with cardiac embolic diseases, giant cell arteritis or systemic vascular inflammations. Our case is the second reported case in English literature of bilateral CRAO following head trauma.

A 30-year-old male presented at our outpatient department (OPD) with complaints of sudden onset bilateral severe loss of vision following head injury, six days back. When the patient was working at a construction site he fell from a height six days ago and the left side of his face slammed on the ground. The patient lost consciousness for a few minutes and was rushed to the general emergency. At emergency it was found that he had an altered consciousness (Glasgow Coma Scale E4 V3 M6), fracture of angle of left mandible [Fig. 1], a few bruises over the left cheek and a subconjunctival hemorrhage in the left eye. No peri-ocular changes or orbital emphysema was noted. X-ray skull and computed tomography (CT) scan of the brain, taken at the emergency did not reveal any abnormality except mandibular fracture. After primary evaluation, he was admitted to the trauma ward. A few minutes after the admission, approximately 2 h after the initial injury, the patient developed sudden severe vision loss in both eyes.

**Figure 1:**
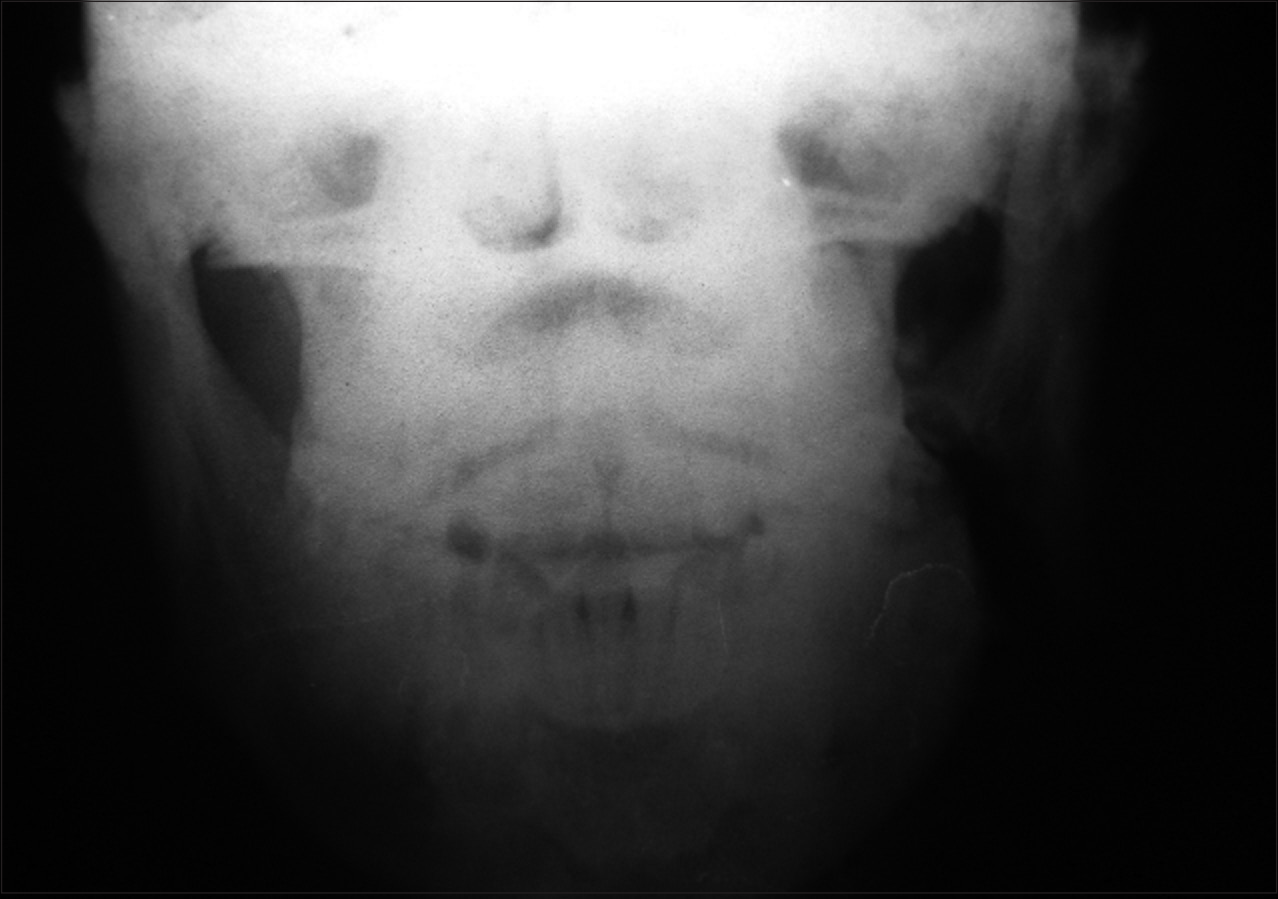
X-ray skull showing fracture of mandible left side

He did not have a relevant past ocular or medical history and his family and social history were noncontributory.

On examination, his vision was finger counting close to the face in right eye and hand movement in left eye, both of which did not improve with pinhole. Projection of rays (PR) was accurate in both the eyes. External and anterior segment evaluation revealed sluggishly reacting pupil in both eyes and subconjunctival hemorrhage in left eye. On fundus examination there were bilateral white out retina with cherry red spots at the fovea and a few superficial retinal hemorrhages [Figs. 2 and 3]. His systemic examination was normal with no features of neurodeficiency. Fundus fluorescein angiography (FFA) showed a delay in arteriovenous transit time (14 sec), normal choroidal filling and narrowed and blocked arterioles at perifoveolar regions of both eyes [Figs. 4 and 5]. No ocular coherence tomography (OCT) or any electrophysiological tests (ERG, EOG) were done. With these clinical features, a diagnosis of bilateral CRAO was made and patient was investigated for presence of any cardiovascular defect (ECG, echocardiography, Doppler of carotid), hypercoagulable state (bleeding time, clotting time, complete hemogram with platelet count, fasting homocystine level, blood sugar, lipid profile) or vasculitis (ESR, C-reactive protein, C-ANCA for Wegner’s granulomatosis, P-ANCA for polyarteritis). None of the investigations revealed any abnormality. CT angiography, protein S and C deficiency, and anti thrombin 3 level were not done. The results of all the tests are given in Table 1.

**Table 1:** Results of the different tests

ECG, Echocardiography, Doppler of carotid	No abnormality detected
Bleeding Time-	5 Min 13 Sec
Clotting time-	2 Min 5 Sec
Complete hemogram-	Hb%-11 gm%
	Normocytic Normochromic
	N62,L29,M9,E0,B0
	Platelets- adequate
ESR	24 mm in 1^st^ h
Fasting homocytine level (Total)-	10.5 micromol/ml
Fasting blood sugar-	86 mg%
Postprandial blood sugar-	142 mg%
Lipid profile-	Cholesterol-204 mg/dl
	Triglyceride- 112 mg/dl
	LDL-94 mg/dl
	HDL-72 mg/dl
C reactive protein-	2.8 mg/dl
C-ANCA	Negative
P-ANCA	Negative

**Figure 2:**
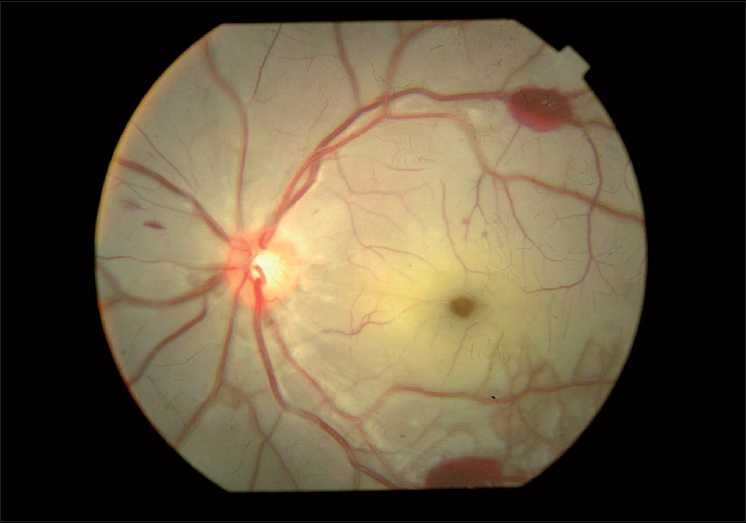
Left eye, white out retina and cherry red spot with few intraretinal hemorrhages

**Figure 3:**
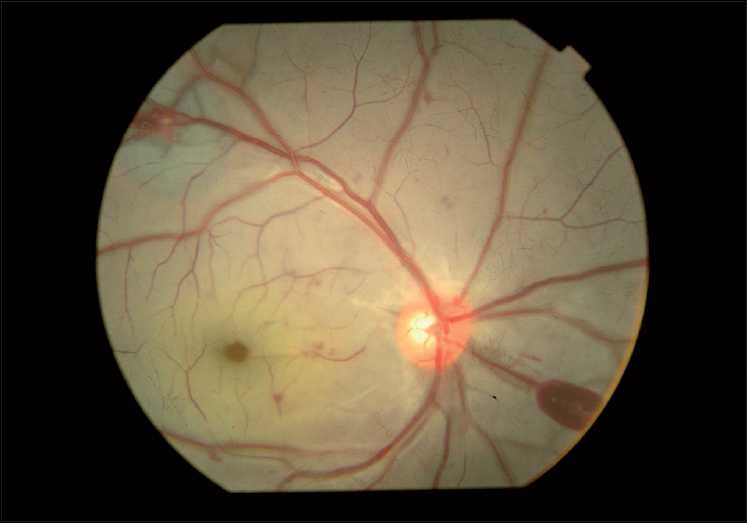
Right eye, white out retina and cherry red spot with few intraretinal hemorrhages

**Figure 4:**
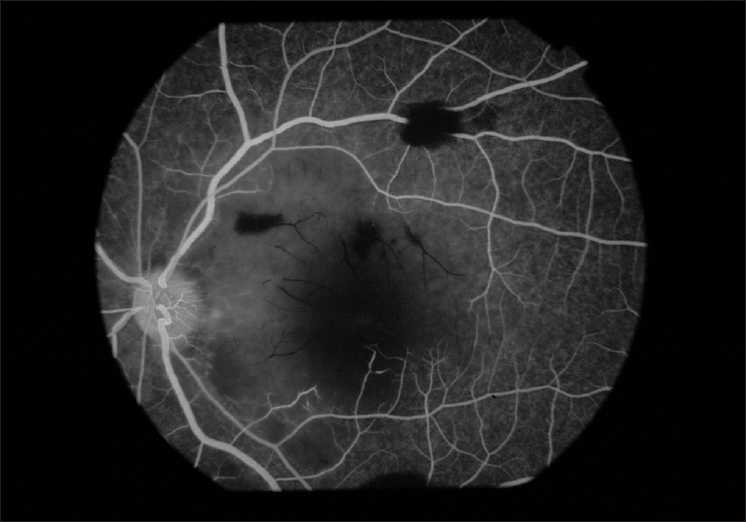
FFA of left eye showing blocked arteriole at perifoveolar region

**Figure 5:**
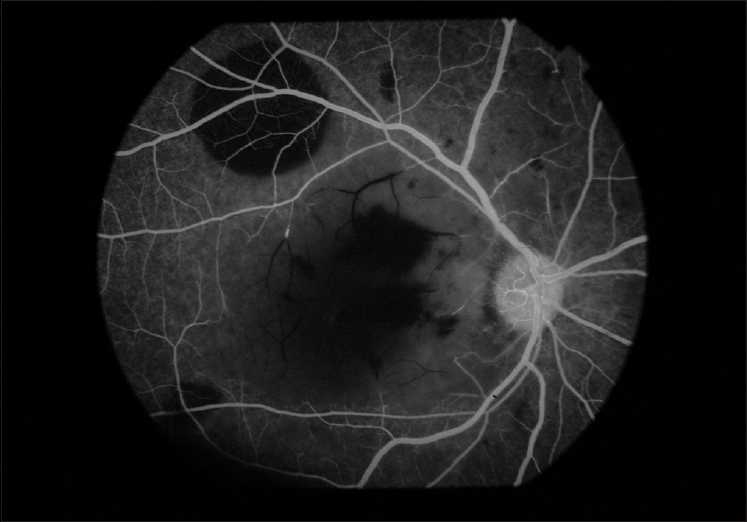
FFA of right eye showing blocked arteriole at perifoveolar region

As the patient presented late in the course of the disease, he was given only high dose of oral multivitamins for one month. He was then followed up atirst month and then sixth month during which no improvement of vision was noted. But during that time the white out retina regained its normal color.

## Discussion

CRAO is a rare event found in 1 in 10,000 outpatient visits. Bilateral involvement is even rarer, found in only 1-2% of total cases.[1]

First described by Von Graefe in 1859 as an embolic event in a patient of endocarditis, CRAO cases are usually associated with thrombus formation at or just proximal to the lamina cribrosa. Only 20-25% cases are associated with embolization and very few cases may be of inflammatory origin like vasculitis or optic neuritis.

Most of the cases present with painless sudden persistent loss of vision in the range of counting fingers to perception of light. A history of amurosis fugax lasting up to 2 h may be present. Anterior segment evaluation is usually normal except for the presence of an afferent pupillary defect. Initially, fundus may appear relatively normal. Eventually, hypoxia results in ischemic whitening of the retina, most pronounced at the posterior pole. A cherry red spot is typical and found in about 90% of cases. Box carting of vessels may be seen in severe obstruction. Splinter hemorrhage is also a common finding but extensive hemorrhage is rare. Within four to six weeks, the retinal whitening usually resolves and optic disc pallor sets in. Macular retinal pigment epithelial changes also start to appear.

Fundus fluorescein agiography typically shows delay in arteriovenous transit time (>11 sec), and retinal arterial filling with arterial narrowing or obstruction. Choroidal filling is usually normal.

No treatment modality has been proven to be effective in CRAO. However, the following measures may be efficacious in improving vision if instituted within 90 to 120 min of occlusion. These include immediate ocular massage and lowering of intraocular pressure by means of anterior chamber paracentesis or drugs (acetazolamide 500 mg i.v or orally, topical beta blocker). In spite of these modalities most patients fail to regain any useful vision.

Bilateral CRAOs were reported in the setting of Wegener’s granulomatosis, temporal arteritis, homocystenuria, sickle cell disease, Henoch-Schonlein purpura, mitral valve prolapse, atherosclerosis and migraine.[2–6] Only one case of bilateral CRAO following head trauma has been reported previously(MEDLINE search http://www.nlm.nih.gov/medlineplus/).[3] Our case is the second reported case in English literature of bilateral CRAO following head trauma. In our case the most likely etiology of obstruction was embolism (clot/fat) from the fractured mandible.
